# Radiation Therapy in the Management of Extensive Giant Condyloma Acuminata With Rectal Involvement: A Case Report

**DOI:** 10.7759/cureus.56882

**Published:** 2024-03-25

**Authors:** Shaveena Sivapalan, Bailey A Loving, Siddharth Ramanathan, Casey P Schukow, John M Robertson

**Affiliations:** 1 Radiation Oncology, Michigan State University College of Osteopathic Medicine, East Lansing, USA; 2 Radiation Oncology, Corewell Health, Royal Oak, USA; 3 Radiation Oncology, Oakland University William Beaumont School of Medicine, Rochester Hills, USA; 4 Pathology, Corewell Health, Royal Oak, USA

**Keywords:** refractory treatment, non-cancerous pathology, hiv, anorectal area, multidisciplinary approach, malignant transformation, human papillomavirus, radiation therapy, rectal involvement, giant condyloma acuminata

## Abstract

Giant condyloma acuminata (GCA) is a rare, locally aggressive manifestation of human papillomavirus (HPV) infection, typically affecting the anorectal area. Patients with GCA often have a poor prognosis due to the high risk of malignant transformation. In this case report, we present a 39-year-old man with HIV who developed progressive and refractory anorectal GCA. Despite initially non-cancerous pathology results, there were concerns regarding a malignant component to the mass. Multidisciplinary discussions led to the decision to pursue definitive radiation therapy. This case report and review of the literature highlight the role of radiation in the management of GCA and the importance of a multidisciplinary approach in the treatment of complex cases.

## Introduction

First described in 1924 by Abraham Buschke, giant condyloma acuminata (GCA), also known as Buschke-Lowenstein tumor, is a rare, locally aggressive variant of condyloma acuminata. While GCAs resemble both common condylomas and squamous cell carcinoma (SCC), these aggressive tumors are unique due to their benign histology, aggressive growth, malignant transformation, and high rates of recurrence after excision [1]. Typically arising in the anogenital region as persistent condyloma acuminatum, the progression to GCA may take three to seven years [2]. Over half of GCAs undergo malignant transformation, commonly followed by rapid infiltration of adjacent structures, with little histological distinction from benign condylomas [1,3]. These behavioral and histological features result in the high morbidity and mortality associated with GCAs, supporting the need for aggressive management strategies [2].

Traditionally, local resection is the standard of care. However, local invasion into perineal structures can complicate operative intervention and may necessitate the use of neoadjuvant therapies to shrink the tumor preoperatively. In locally advanced cases, surgery may not be a feasible option. In such cases, given the high malignant potential of GCA, it is reasonable to pursue a treatment paradigm similar to the management anal cancer. This would involve treating the mass definitively with radiation therapy, typically in combination with a radiosensitizing chemotherapy agent to achieve local control and improve patient outcomes. Although it is well known that concurrent chemotherapy improves local control and survival in patients with anal cancer, both acute and late toxicity are significantly higher with this treatment approach [4]. In patients with HIV, this may be of particular concern due to compromised immune function. Therefore, radiation therapy alone may be considered to be a balanced treatment approach in this patient population.

We report a case of a patient with HIV infection and a history of recurrent anal condylomas, who developed locally advanced GCA. Given the aggressive infiltration of the tumor into adjacent structures, this patient required a unique management strategy utilizing definitive radiotherapy alone.

## Case presentation

A 39-year-old man with a longstanding history of HIV/AIDS on highly active antiretroviral therapy (HAART) of Biktarvy once a day, presented to his infectious disease specialist for routine follow-up. The patient's CD4 count was observed at 288, indicating an improvement from the previous year's count of 130. He had been experiencing two years of recurrent anal fistulas, requiring two fistulotomies eight and nine months prior to presentation.

**Figure 1:**
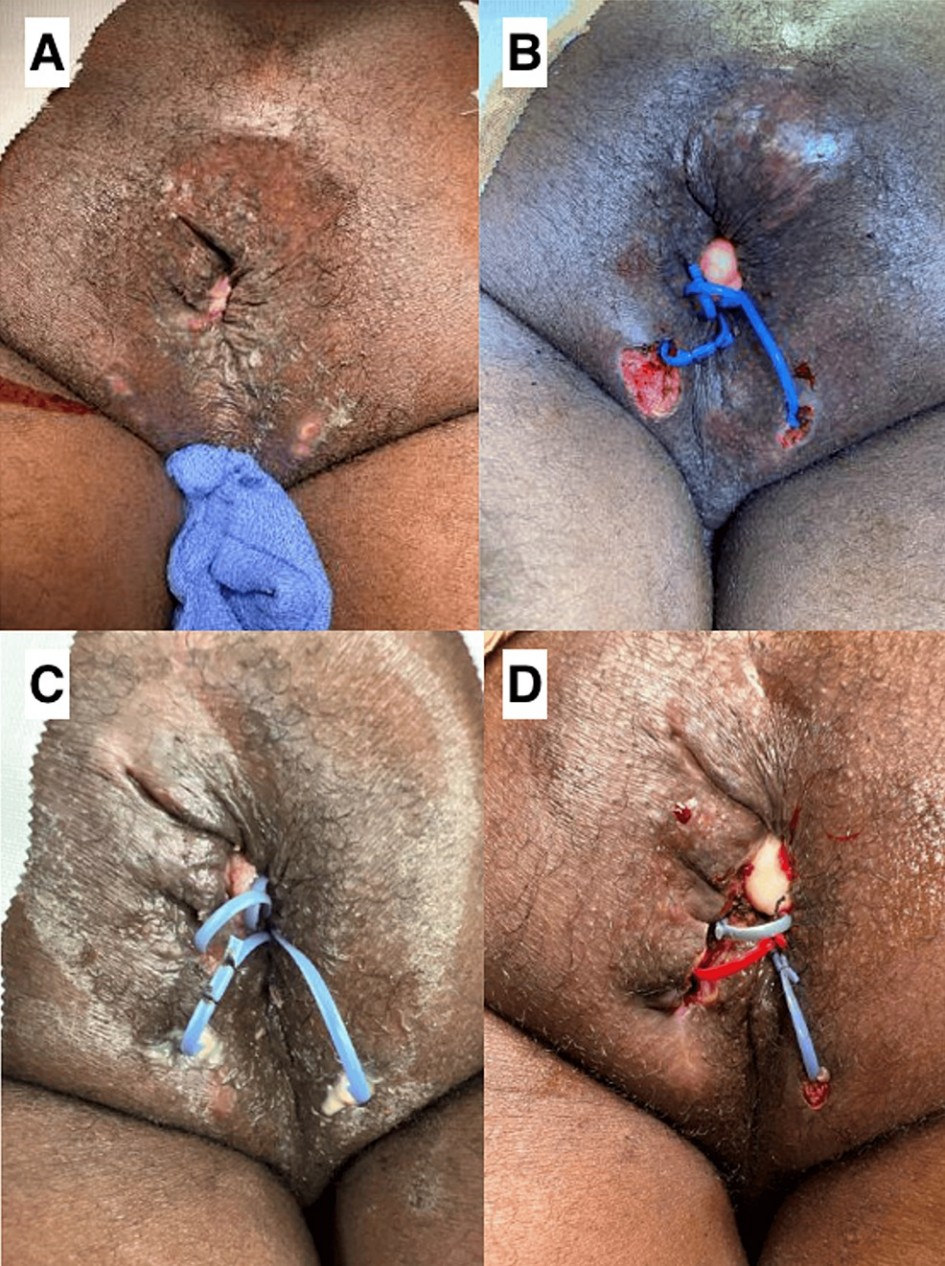
Appearance of anus following first and second fistulotomies with condyloma fulguration. (A) The pre-operative appearance of the anal region prior to the first anal examination under anesthesia (EUA). (B) The post-operative appearance of the anal region following condyloma excision and seton placement during EUA. (C) The pre-operative appearance of the anal region prior to the second anal EUA (six months following the first condyloma excision). Pus originating from anus was noted; consistent with re-opening/drainage of prior abscess from first EUA. (D) The post-operative appearance of the anal region following the second EUA. The patient required a second fistulotomy with further condyloma fulguration and new seton placement.

The patient expressed frustration with persistent rectal pain and anogenital warts and was referred to a general surgeon, who performed a colonoscopy. Advancement of the colonoscope was impeded by a large, pedunculated, partially obstructing anorectal mass. The mass was biopsied and the pathology was consistent with a low-grade squamous intraepithelial lesion (anal intraepithelial neoplasia grade I (AIN I)). A rectal examination under anesthesia (EUA) identified multiple abscesses and a recurrent fistula and scant stool in the rectal vault. The patient underwent fulguration of anal lesions, drainage of multiple abscesses, and with a seton placement (Figure 1). The patient was prescribed topical Imiquimod to be applied every other day prior to bedtime for the post-surgical management of the resected condylomas.

The patient was initially scheduled for a three-month follow-up visit with the general surgeon; however, this appointment was postponed because the patient required emergency surgery for a stab wound in their left flank.

**Figure 2:**
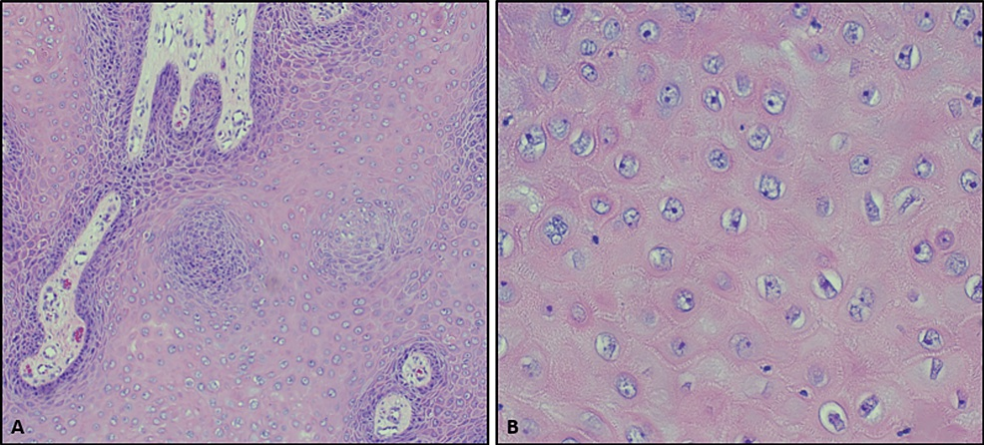
H&E of tissue fragments from patient's anus. Acanthosis, or thickening of the stratum spinosum, is present in the left-hand image (A, 10x), while koilocytosis (irregular nuclei, multinucleation, and perinuclear vacuolization) is noted in the right-hand image (B, 40x). These histologic findings in the patient's clinical context, along with negative p16 block-positive IHC staining (not shown) and absence of definitive high-grade dysplasia, support a diagnosis of GCA. GCA: giant condyloma acuminatum; H&E: hematoxylin & eosin; IHC: immunohistochemistry

A follow-up visit at six months revealed several recurrent and unresolved condylomas. The patient underwent a second colonoscopy and EUA for condyloma re-excision. The biopsy specimens obtained during the colonoscopy were insufficient, so repeat biopsies were conducted during the EUA. Six fragments of varicoid skin were fixed with formalin and sent to pathology, along with samples of the left anterior fistula tract and an anterior skin tag. Histological examination of these fragments was remarkable for acanthosis and koilocytosis (irregular nuclei, multinucleation, and perinuclear vacuolization; Figure 2). Immunohistochemistry of this tissue was negative for p16 block-positive staining (not shown) and negative for definitive high-grade dysplasia, supporting a diagnosis of GCA. Immunohistochemistry of this tissue found that the tumor was negative for block-positive staining in A2-2 and negative for definitive high-grade dysplasia in C2-2 antibodies (Figure 2). Following the procedure, he reported difficulty urinating and significant perianal pain that radiated to the left groin.

**Figure 3:**
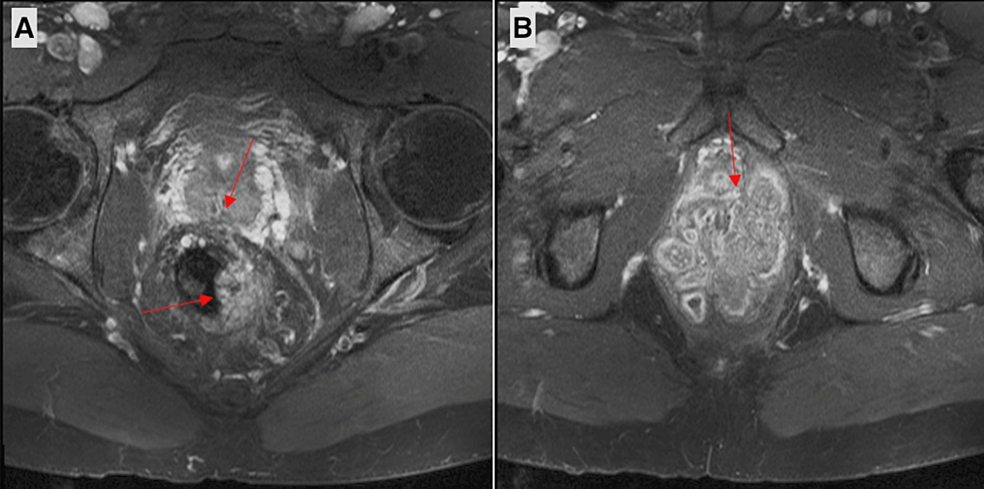
Axial T2-weighted MRI images of the pelvis before radiation therapy showing rectal involvement of the mass at inferior arrow and invasion into the periprostatic space at superior arrow (A) and infiltrating the base of the penis (B).

A two-month post-operative gadolinium-enhanced MRI revealed a 7.5 cm lobulated cauliflower-like mass extending into the mesorectal and periprostatic space, with invasion of the corpus spongiosum of the penis, and mild enlargement of several pelvic lymph nodes (Figure 3). Visual features of the mass and the degree of intraluminal obstruction raised concern for an underlying SCC.

Treatment

**Figure 4:**
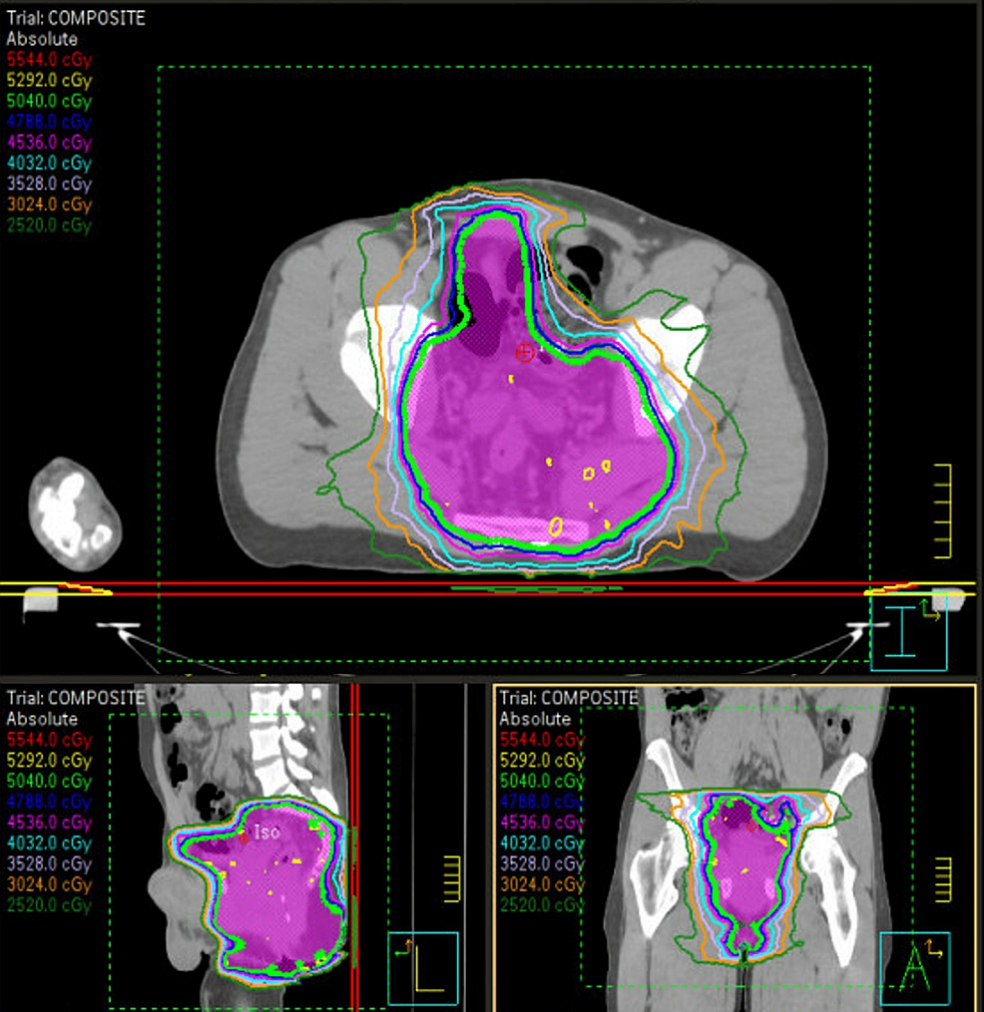
Patient radiation therapy treatment plan Legend: The isodose lines correlating with the respective colors are displayed in the top right-hand corner of the figure. The planning target volume (PTV) is displayed in a magenta color.

**Figure 5:**
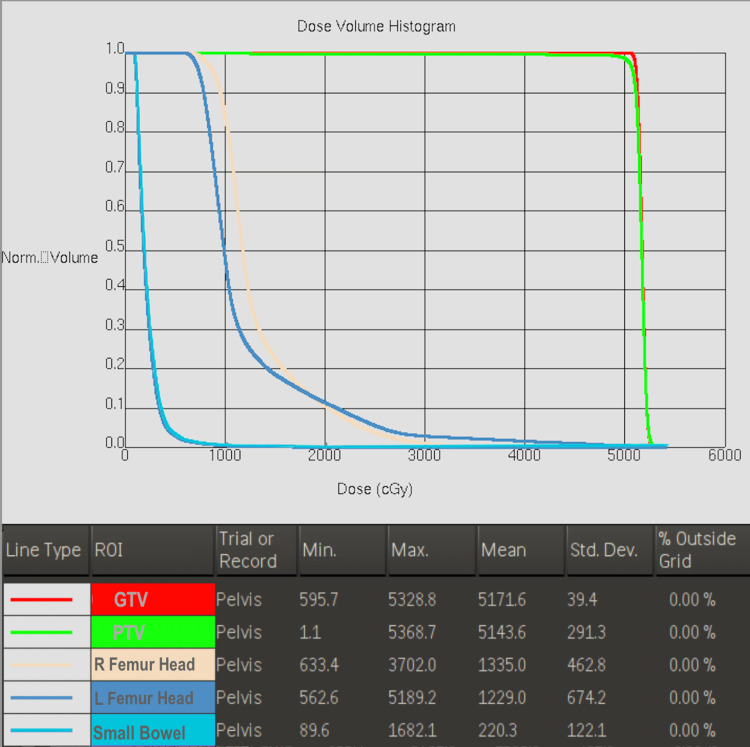
Dose-volume histogram of organs at risk in patient’s radiation treatment plan as detailed in Figure 4, compiled using MIM software (MIM Software Inc., Cleveland, OH, USA).

This case was presented to a multi-disciplinary colorectal tumor board, and a consensus was made to refer the patient to the radiation oncology department for consideration of definitive radiation therapy. Given the patient’s poor response to condyloma excision and adjuvant imiquimod and the alternative of a total exenteration, definitive radiotherapy was considered the most conservative treatment option [5]. The risks and benefits of radiotherapy were discussed with the patient, including the risk of infertility. Given the patient’s desire to preserve his fertility, a clamshell was used throughout the treatment. A total of 50.4 Gy was delivered to the pelvic region in 28 fractions. The contours, field design, and dose constraints were in concordance with the Radiation Therapy Oncology Group (RTOG) 0529 standard for the treatment of anal cancer (Figure 4) [5]. All dose constraints were met (Figure 5).

Outcomes and follow-up

The patient tolerated treatment well, without interruptions. Aside from acute Common Terminology Criteria for Adverse Events (CTCAE) grade 1 diarrhea, he also developed acute CTCAE grade 2 moist desquamation towards the end of treatment, which required aluminum sulfate tetradecahydrate (Domeboro® (Bayer HealthCare LLC, Whippany, USA)) soaks four times a day. Despite detailed follow-up instructions, the patient failed to present for a three-month follow-up CT of the abdomen and pelvis, and has been unreachable by phone due to phone service termination since the completion of treatment. This may be explained by the patient’s current homelessness, as relayed to the colorectal surgeon. The patient has followed up with general surgery six months following radiotherapy and reported that the patient is “feeling the best he has felt in a very long time since completion of RT." He denied any treatment-related toxicity at this visit. Perianal exam revealed the presence of chronic CTCAE grade 1 radiation-induced induration. Anoscopy showed a complete clinical response, noting no condylomas on the exam.

## Discussion

The odds of developing GCA in the perianal region are increased in cases of immunosuppression, tobacco use, and early sexual intercourse [6]. The patient’s diagnosis of HIV and known history of tobacco use placed them at a higher risk of development. Within a seven-year time frame, the patient's recurrent anogenital warts developed into an aggressively invasive form of GCA. Given the locally aggressive nature of the GCA in this patient, paired with the proximity to vital structures, non-surgical treatment was necessary to preserve the quality of life.

Wide local excision or abdominal perineal resections are broadly considered the treatments of choice for patients presenting with GCA. According to a review of 42 GCA cases, up to 56% of those such cases malignantly transformed into SCC [1].

Several alternative treatment options have been explored in the literature. Generally, these conservative options have shown higher rates of local recurrence, and no singular treatment has been proven to achieve satisfactory disease control [7].

GCAs are often initially managed with topical therapies including imiquimod, podophyllotoxin, and sinecatechins. More commonly prescribed for the treatment of HIV-induced anogenital warts, these topical treatments are utilized as adjuvant therapies to more invasive therapies in the management of GCA [8,9]. As observed in our patient, who was prescribed topical imiquimod every other night, topical therapies alone have not proven to be effective in achieving total regression of condylomas. There has been a singular case of complete regression of vulvar GCA with no recurrence after three years [10].

Laser-based therapies are a better treatment option for GCA. Photodynamic therapy (PDT), which engages a photodynamic reaction to target selective tissues, has been largely successful in the treatment of classic condyloma acuminata. When combined with oral acitretin and aminolevulinic acid (ALA), patients experienced a 94% cure rate [11]. When applied to GCA, PDT has yielded more ambiguous outcomes. While PDT has completely eradicated the underlying GCA in some patients [12], in others PDT resulted in a reduction of GCA symptoms without a significant reduction in lesion size [13].

Although CO2 lasers have been historically successful in managing verrucous carcinoma [8], CO2 laser vaporization is reserved for cases of surface level, non-penetrating GCAs, post-operative ablation, and recurrent GCA following surgical intervention [14].

Cryotherapy offers another minimally invasive treatment option for patients with GCA. Cryotherapy has been successfully utilized for decades to achieve complete local control in patients with GCA [15]. However, the requirement of combination therapy in patients with locally advanced disease serves as a limitation to broader use [16]. Furthermore, cryotherapy imposes a significant burden on the patient, as the frequency with which cryotherapy must be conducted exceeds that of all laser and topical therapies.

The most extensively studied alternative to surgical excision is chemotherapy. While there have been cases of anogenital verrucous carcinomas successfully treated with systemic chemotherapy, the existing data on GCAs only pertains to smaller, superficial lesions [17]. For local control of larger lesions, the use of chemotherapeutics is focused on reducing the tumor burden in the pre- and postoperative settings [18,19]. Oral chemotherapy with fluorouracil (5-FU) as neoadjuvant therapy is largely effective when combined with local surgical excision and radiation therapy [20,21]. However, while concurrent chemotherapy and radiotherapy regimens traditionally used to treat anal SCC may be effective in patients with GCA, there remains a need for a more thorough investigation to evaluate the potential efficacy and long-term treatment safety of this treatment approach [18]. For GCAs that remain refractory to chemoradiation, surgical intervention through an abdominoperineal resection (APR) is warranted.

Through a multidisciplinary tumor board for discussion of management options, surgical resection was confirmed to be limited due to the lack of clear planes between the mass and adjacent structures. Wide local excision would be near impossible without excision of portions of the patient's lower left penis, prostate, and perianal region. While flap reconstruction is possible for some patients [22], radiotherapy was deemed a more suitable treatment choice due to age and quality of life. The unique management outlined in this case report has been utilized in 11 other reported cases of locally advanced GCA in a literature review spanning over 30 years (Table 1).

**Table 1:** A literature review of published cases with GCA treated with radiation therapy spanning from 1987 to 2019. GCA: giant condyloma acuminata, HPV: human papillomavirus, 5FU: fluorouracil, SCC: squamous cell carcinoma, APR: abdominoperineal resection

First Author/Pub Year	Gender	Age	HPV/HIV	Therapy	Fractions	Follow-Up	Recurrence	Additional Therapy	Pathology
Butler, 1987 [18]	Male	40	N/A	Definitive Radiotherapy, 5FU, mitomycin	45Gy (28 fractions over 44d)	32w	No	Diverting colostomy, surgical excisions	GCA w/ marked acanthosis, hyperkeratosis, and papillomatosis with well-differentiated in situ and invasive SCC
Hyacinthe, 1998 [23]	Male	60	HPV+ (6/11/16/18/31/33/35)	Radiotherapy, 5FU, mitomycin-C, posterior pelvic exenteration	46.8Gy (26 fractions over 46d)	2y	No	No	GCA with SCC transformation
Sobrado, 2000 [24]	Male	42	N/A	Definitive Radiation therapy	45 Gy (28 fractions) during additional radiation therapy	20mo	No	PRIOR to Radiation Therapy: Sigmoidostomy, bleomycin, topical podophyllin (recurrence in <30d)	
Dolanc, 2002 [25]	Female	56	HPV+ (6/11)	Radiotherapy, APR (Abdominoperineal Resection)	50Gy			No	SCC arising in GCA w/ clear resection margins
Chao, 2005 [26]	Male	57	HIV-	Radiotherapy, 5FU, mitomycin	50.4Gy to primary tumor, 45Gy to perirectal LN, 36Gy to inguinal LN	1y	No	No	
Tytherleigh, 2006 [27]	Male	40	HIV+	chemoradiation, APR (Abdominoperineal Resection)		12mo	Yes, patient passed	N/A	GCA
	Male	51	HIV+	chemoradiation, APR (Abdominoperineal Resection)		5y	No	N/A	GCA
Armstrong, 2009 [28]	Male	46	HIV-, HPV+ (6/11)	Unsuccessful APR, chemoradiotherapy		34mo	No	No	GCA
Handisurya, 2009 [29]	Male	45	HIV+, HPV+ (6/11)	Surgical intervention	60Gy (additional radiation therapy)	6mo	Yes	Palliative surgery and chemoradiotherapy	GCA differentiating into invasive SCC
Haque, 2009 [30]	Male	38	HIV-	Definitive Radiotherapy, 5FU, mitomycin	54Gy (30 fractions)	6mo	No	PRIOR to Radiation Therapy: Two Surgical excisions (recurrence in <5mo)	GCA (Buschke-Lewenstein tumor-type) w/ focal transformation to verrucous carcinoma
Indinnimeo, 2013 [21]	Male	43	HIV+, HPV+ (6)	Radiotherapy, 5FU, mitomycin-C	45Gy (28 fractions) to the pelvis plus a boost with 14.40Gy (1.8Gy/fr) to the primary tumor	3y	No	EUS and high-resolution anoscopy (HRA)	GCA with SCC transformation
Shenoy, 2019 [31]	Male	70	HIV-, HPV+	Radiotherapy, 5FU, mitomycin	46Gy (30 fractions)	3y	No	No	GCA, SCC of the anus
	Male	58	HIV+, HPV+	Radiotherapy, 5FU, mitomycin, diverting sigmoid colostomy	46Gy (30 fractions)	3mo	Yes	No	GCA, SCC of the anus

The patient underwent a course of external beam radiation therapy delivering 50.4 Gy in 28 fractions. While concurrent chemotherapy was also considered to enhance treatment efficacy, the decision was made to treat with radiation alone. The patient was informed about the potential acute and long-term side effects of radiation therapy and presented with fertility preservation options.

While wide surgical excision would be the standard of care and result in the greatest chance of survival, the patient would be required to have an ostomy for the remainder of their life which would necessitate total reconstruction of the penis. Given the patient's standing as a relatively healthy 39-year-old male, excluding HIV and GCA diagnoses, preserving the patient’s quality of life was deemed a priority.

At six months follow-up with general surgery, anoscopy reflected complete regression of condylomas in this patient. Given the nature of GCA to undergo malignant transformation [1], a complete clinical response is vital to preventing further recurrence or progression of the cancer. Based on the literature review conducted on those cases utilizing radiation therapy as a definitive treatment option, primarily after the failure of more common treatment options, only 15% experienced recurrence. The patient's positive clinical outcome could be correlated to a decreased probability of recurrence and/or progression of disease to a malignant state.

GCA with rectal involvement poses significant challenges in terms of treatment planning and management decisions. The case report emphasizes the role of radiation therapy in the multimodal management of this condition, especially when surgical options are limited. A comprehensive and multidisciplinary approach is crucial in determining the optimal treatment strategy and addressing patient concerns. While further studies are warranted to evaluate the efficacy and long-term outcomes of radiation therapy in this rare and aggressive disease, for this patient, the adequacy of radiation therapy in the treatment of GCA is evident through the patient’s total regression of disease and most notably through the patient’s improved quality of life.

## Conclusions

In summary, this case report details a progressive and treatment-resistant GCA, emphasizing the challenges associated with its management. The patient's anorectal GCA, refractory to traditional surgical and topical treatments, raised concerns for possible malignant transformation. After a thorough multidisciplinary evaluation, definitive radiation therapy was pursued, resulting in a complete clinical response and significant improvement in the patient's quality of life. This case supports radiation therapy as a viable treatment option for GCA, particularly in complex cases where surgical interventions fail and malignancy is a concern.
